# Expression Profiles of Long Noncoding RNA and mRNA in Epicardial Adipose Tissue in Patients with Heart Failure

**DOI:** 10.1155/2019/3945475

**Published:** 2019-07-04

**Authors:** Meili Zheng, Lei Zhao, Xinchun Yang

**Affiliations:** Heart Center, Beijing Chao-Yang Hospital, Capital Medical University, Beijing, China

## Abstract

The expression profile of long noncoding RNA (lncRNA) in human epicardial adipose tissue (EAT) has not been widely studied. In the present study, we performed RNA sequencing to analyze the expression profiles of lncRNA and mRNA in EAT in coronary artery disease (CAD) patients with and without heart failure (HF). Our results showed RNA sequencing disclosed 35673 mRNA and 11087 lncRNA corresponding to 15554 genes in EAT in total, while 30 differentially expressed lncRNAs (17 upregulated and 13 downregulated) and 278 differentially expressed mRNAs (129 upregulated and 149 downregulated) were discriminated between CAD patients with and without HF (*P*<0.05; fold change>2); lncRNA ENST00000610659 drew specific attention for it was the top upregulated lncRNA with highest fold change and corresponded to UNC93B1 gene, which was proved to be related to HF and encoded UNC93B1 protein regulating toll-like receptor signaling, and both of them significantly increased in HF patients in qRT-PCR validation; the top significant upregulated enriched GO terms and KEGG pathway analysis were regulation of lymphocyte activation (GO:0051249) and T cell receptor signaling pathway (hsa04660), respectively. The current findings support the fact that EAT lncRNAs are involved in the inflammatory response leading to the development of HF.

## 1. Introduction

Recent studies have reported on long noncoding RNA (lncRNA) expression profiling in various human tissues [1]; however, expression profile of lncRNA in human epicardial adipose tissue (EAT) has yet to be described in detail. It is known that a large proportion of the mammalian genome is transcribed as lncRNA, which resides within or between coding genes. In addition, many lncRNAs have been shown to be functional and involved in specific physiological and pathological processes, through transcriptional or posttranscriptional regulatory mechanisms [2, 3]. To date, however, lncRNAs have never been included in analyses of the human EAT transcriptome. EAT is a key cardiometabolic factor, where, by releasing various inflammatory factors [4], EAT can modulate cardiac function and correlate with heart failure (HF) [5, 6], independently of metabolic status or the presence of coronary artery disease (CAD).

In the present study, we sought to supplement EAT lncRNA and mRNA expression profiles to provide a more complete picture of the myocardial transcriptional landscape in heart failure and also provide possible biomarkers for HF.

## 2. Materials and Methods

### 2.1. Study Participants

EAT samples were taken from 10 CAD patients who underwent coronary artery bypass graft surgery, in the Department of Heart Center, Beijing Chao-yang Hospital of Capital Medical University. Subjects were divided into two groups: HF group (n=5) and non-HF group (n=5). HF group included patients with Brain Natriuretic Peptide (BNP)>500ng/L and abnormal echocardiography finding (left ventricular end diastolic diameter [LVEDD]>50mm in female and >55mm in male and left ventricular ejection fraction [LVEF]<50%); non-HF group included patients with BNP<100 ng/L and normal views in echocardiography. The protocol was approved by the Ethics Committee of Beijing Chao-yang Hospital affiliated with Capital Medical University and written informed consent was obtained from participants before the study.

### 2.2. RNA Sequencing Procedure

Total RNA was extracted from the EAT and quantified using a NanoDrop ND-1000 instrument. 1-2*μ*g total RNA was used to prepare the sequencing library in the following steps: (1) Total RNA is enriched by oligo (dT) magnetic beads (rRNA removed). (2) Using KAPA Stranded RNA-Seq Library Prep Kit (Illumina), RNA-seq library preparation incorporates dUTP into the second cDNA strand and makes the RNA-seq library strand-specific. (3) After completing, libraries were qualified with Agilent 2100 Bioanalyzer and quantified by absolute quantification qPCR method. (4) Sequence the libraries on the Illumina HiSeq 4000 instrument (we followed the methods of Wang et al. 2019 [7]).

### 2.3. Quantitative RT-PCR

qRT-PCR was used to measure selected lncRNA ENST00000610659 and UNC93B1 mRNA. Total RNA samples were extracted from the EAT samples using TRIzol (Invitrogen, Carlsbad, CA). The relative expression levels of mRNA and lncRNA were quantified using ViiA 7 Real-Time PCR System (Applied Biosystems, Foster City, USA) according to standard methods. lncRNA ENST00000610659: the forward primer was 5′ CGGCTTCAACAAGACGGTTC 3′, the reverse primer was 5′ AAGGCTCCACTCCGCACAAA 3′; UNC93B1 mRNA: the forward primer was 5′ GCTCACCTACGGCGTCTACC 3′, the reverse primer was 5′ CGGTAGGTCTCGT CGTAGTGC 3′.

### 2.4. Statistical Analysis

R package was used to calculate the FPKM value and differential expression for gene and transcript level and perform hierarchical clustering, GO enrichment, pathway analysis, scatter plots, and volcano plots with the differentially expressed genes. Descriptive statistics for each variable were determined. Continuous variables were expressed as the mean ± SD and compared using unpaired Student's t-test, and categorical variables were expressed as percentages and numbers and were compared using the chi-squared test. Significant GO enrichment and pathways were selected by Fisher's exact test, and p<0.05.

## 3. Results

### 3.1. Characteristics of Participants

The present study comprised 10 CAD patients (5 with HF and 5 without). The main clinical characteristics of the two groups are summarized in Table 1. There were no significant differences in subject characteristics between the two groups; they were well balanced with regard to main clinical and laboratory characteristics. The CAD patients with HF had higher BNP level and LVEDD and lower LVEF.

### 3.2. RNA Sequencing Data

Using RNA sequencing, we detected 46760 transcripts (including 35673 protein-coding and 11087 non-protein-coding with linear structure and length>200bp) corresponding to 15554 genes in EAT in total. The top 30 highly expressed protein-coding and non-protein-coding transcripts are summarized in Table 2.

**Figure 1:**
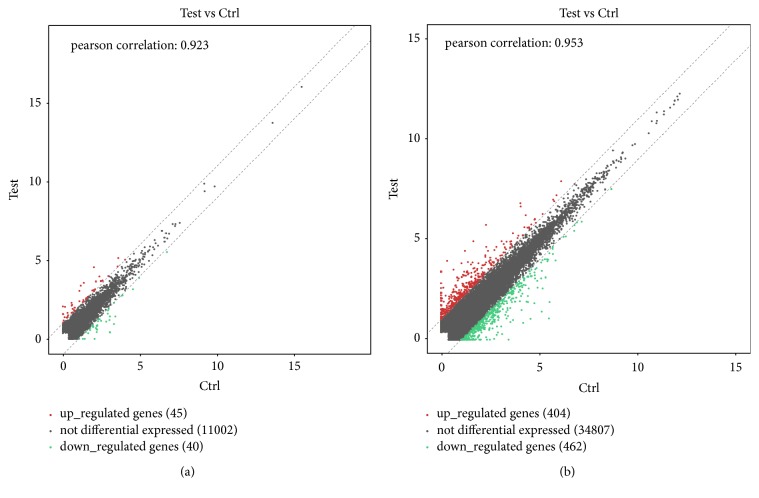
The scatter plot of the differential expressed (p<0.05) lncRNA (a) and mRNA (b) in patients with heart failure (HF) and without heart failure (non-HF) (red or green represented upregulated or downregulated genes, respectively); 85 lncRNA and 866 mRNA were identified, including 45 upregulated and 40 downregulated lncRNA, as well as 404 upregulated and 462 downregulated mRNA.

Scatter plot (Figure 1) was performed to group lncRNA and mRNA and display the levels of lncRNA and mRNA in CAD patients with and without HF according to their expression levels among samples, and the results indicated that the lncRNA and mRNA expression profiles in CAD patients with HF were distinctly different from those in CAD patients without HF. 85 lncRNA and 866 mRNA whose levels changed significantly (p<0.05) were identified, including 45 upregulated and 40 downregulated lncRNA, as well as 404 upregulated and 462 downregulated mRNA.

**Figure 2:**
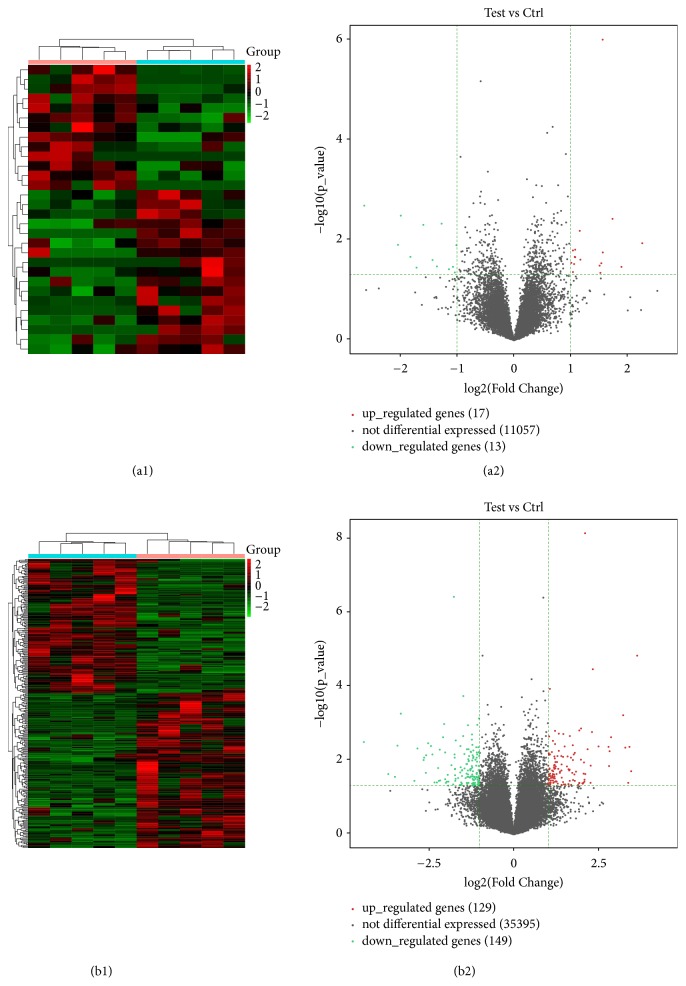
The hierarchical clustering and volcano plot of the substantially differential expressed (P<0.05; fold change>2) lncRNA ((a1) and (a2)) and mRNA ((b1) and (b2)) in patients with and without heart failure (red or green represented upregulated or downregulated genes, respectively); 30 lncRNA and 278 mRNA were identified, including 17 upregulated and 13 downregulated lncRNA, as well as 129 upregulated and 149 downregulated mRNA.

**Figure 3:**
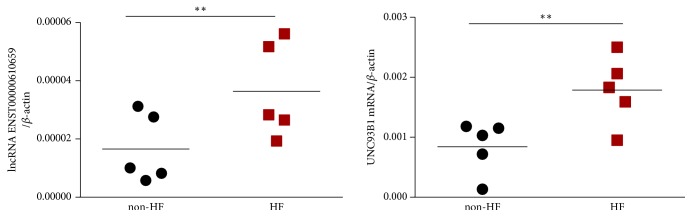
qRT-PCR analysis of expression of lncRNA ENST00000610659 (left) and UNC93B1 mRNA (right) in patients with heart failure (HF) and without heart failure (non-HF) (n=5 in each group), *∗∗*p<0.05.

Using a 2-fold expression difference as a cutoff, a total of 30 differentially expressed lncRNAs (17 upregulated and 13 downregulated) (Figure 2, Table 3) and 278 differentially expressed mRNAs (129 upregulated and 149 downregulated) were discriminated between CAD patients with and without HF (Figure 2). Among them, lncRNA ENST00000610659 was the top upregulated lncRNA with highest fold change and corresponded to UNC93B1 gene, which was proved to be related to HF and encoded UNC93B1 protein regulating toll-like receptor signaling. lncRNA ENST00000610659 and UNC93B1 mRNA were both significantly increased in HF patients in qRT-PCR validation (p=0.040 for lncRNA ENST00000610659 and p=0.019 for UNC93B1 mRNA) (Figure 3). lncRNA ENST00000610659 might be a potential biomarker for HF.

### 3.3. GO and KEGG Pathway Analysis of Differentially Expressed mRNAs

**Figure 4:**
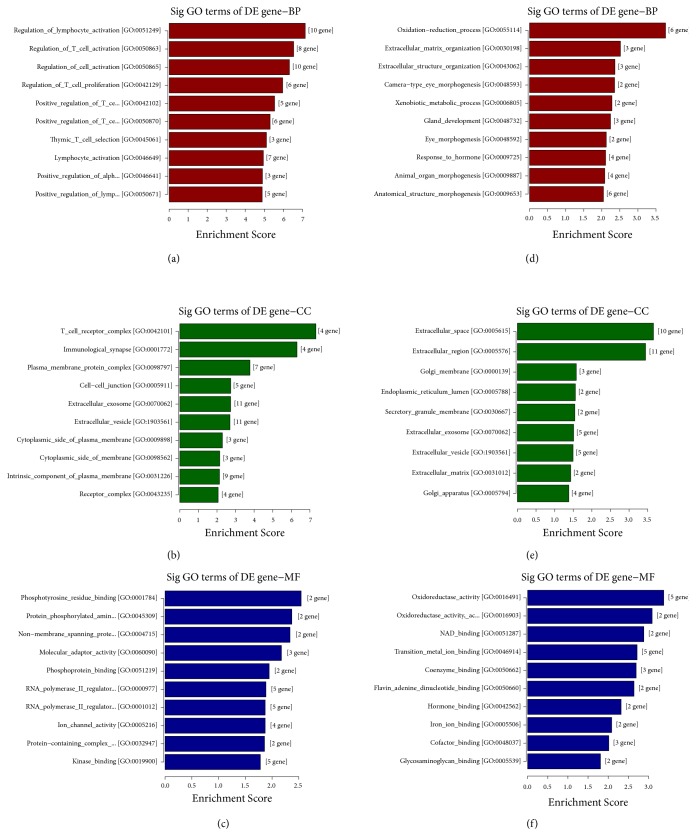
Enriched GO terms analysis for differentially expressed mRNAs. Top 10 significantly upregulated GO terms-Biological Process (a) and Molecular Function (c); all significantly upregulated GO terms-Cellular Component (b); top 10 significantly downregulated GO terms-Biological Process (d) and Molecular Function (f); all significantly downregulated GO terms-Cellular Component (e).

The Gene Ontology (GO) project (Figure 4) provided a controlled vocabulary to describe gene and gene product attributes in any organism. The ontology covered three domains: Biological Process (BP), Cellular Component (CC), and Molecular Function (MF). For upregulated genes, the top enriched GO terms in three domains were regulation of lymphocyte activation (GO:0051249) in BP, T cell receptor complex (GO:0042101) in CC, and phosphotyrosine residue binding (GO:0001784) in MF; for downregulated genes, those were oxidation reduction process (GO:0055114) in BP, extracellular space (GO:0005615) in CC, and oxidoreductase activity (GO:0016491) in MF, respectively.

**Figure 5:**
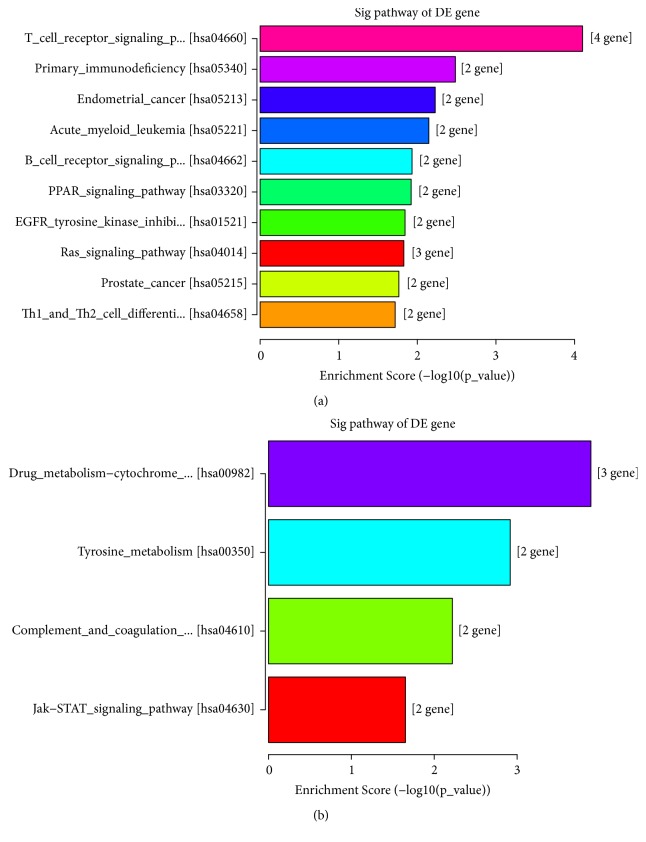
KEGG pathway analysis of differentially expressed mRNAs. Top 10 significantly upregulated pathways (a) and all significantly downregulated pathways (b).

Pathway analysis (Figure 5) showed that, when comparing to controls, 17 pathways were significantly upregulated while 4 pathways were significantly downregulated. The top 3 significantly upregulated pathways were T cell receptor signaling pathway (hsa04660), primary immunodeficiency (hsa05340), and endometrial cancer (hsa05213). Meanwhile, the significantly downregulated pathways were drug metabolism cytochrome P450 (hsa00982), tyrosine metabolism (hsa00350), complement and coagulation cascades (hsa04610), and Jak-STAT signaling pathway (hsa04630).

## 4. Discussion

In the present study, we assessed the expression profiles of EAT lncRNA and mRNA in CAD patients with and without HF. The results showed a total of 35673 mRNA and 11087 lncRNA corresponding to 15554 genes in EAT were detected, and using a 2-fold expression difference as a cutoff, a total of 30 differentially expressed lncRNAs (17 upregulated and 13 downregulated) and 278 differentially expressed mRNAs (129 upregulated and 149 downregulated) were discriminated between CAD patients with and without HF.

The differentially expressed lncRNAs corresponded to genes associated with inflammatory response or other factors which are involved in HF. UNC93B1, the top upregulated gene lncRNA corresponded to, encodes UNC93B1 protein that is involved in innate and adaptive immune response by regulating toll-like receptor signaling [8, 9] and is proved to be related to left ventricular diastolic function, heart failure morbidity, and mortality [10]. RBL2 is related to TGF-beta signaling [11]. LINC00963 encodes lncRNA963 playing an important role in chronic renal failure, which is closely associated with chronic diseases such as congestive heart failure [12]. TRIM52 encodes TRIM52 protein that positively regulates the nuclear factor-kappa B signaling pathway [13]. RPS21 (also known as HLDF) encodes HLDF protein that is involved in the mechanisms of blood pressure regulation [14]. AMOTL2 is required for migration and proliferation of endothelial cells during angiogenesis [15]. FBLN5 protein expression significantly decreases in human aneurysmatic aortas and may mediate cell-extracellular matrix interactions and elastic fibre assembly by inflammation [16]. TMTC1 is associated with the risk of incident HF [17]. LRRFIP1 is associated with adiposity and inflammation [18], and LRRFIP1 protein may regulate platelet function [19].

EAT refers to the fat depot that exists on the surface of the myocardium and is contained entirely beneath the pericardium, which generates various inflammatory factors [20, 21]. Factors released from EAT have vasocrine and paracrine effects on the myocardium contributing to modulating properties on cardiac function [4, 22]. As our study showed, lncRNA can also be released from EAT and may be involved in HF; top upregulated lncRNA in HF corresponded to genes associated with inflammatory response and top upregulated enriched GO terms and KEGG pathway of mRNA were also about inflammatory cells activity.

The present study showed the expression profiles of EAT lncRNA and mRNA in CAD patients and also characterized specific EAT lncRNA expression in HF. The EAT lncRNA may be important effector molecules for cardiovascular disease. Through the paracrine and vasocrine transmission, the EAT lncRNA may diffuse across the interstitial fluid or blood into the myocardium to be involved in the development of HF. Our data supplement lncRNA expression profiles in the EAT for lncRNA identifying in heart tissues and also provide possible biomarkers for HF, and further studies are needed to prove it.

## Figures and Tables

**Table 1:** Participants Characteristics.

	CAD with HF	CAD without HF	P value
	(N=5)	(N=5)	
Male, n(%)	2(40)	3(60)	0.999
Age, years	67.8±5.0	60.8±8.1	0.139
BMI, kg/m^2^	23.8±4.6	25.0±5.5	0.718
Diabetes, n(%)	2(40)	2(40)	1.000
Hypertension, n(%)	4(80)	5(100)	1.000
HbA1c, %	6.85±0.87	7.00±1.81	0.871
Total cholesterol, mmol/L	3.67±3.07	3.60±1.52	0.965
LDL-C, mmol/L	2.34±2.52	2.24±1.46	0.941
HDL-C, mmol/L	0.84±0.53	0.70±0.12	0.580
Triglyceride, mmol/L	0.89±0.38	1.86±0.89	0.055
Creatinine, *μ*mol/L	88.2±25.2	76.8±16.8	0.424
Uric acid, *μ*mol/L	363.2±82.4	357.0±69.3	0.901
BNP, pg/ml	1795.4±1053.3	76.4±22.6	0.006
LVEDD, mm	60.0±5.9	47.5±5.2	0.007
LVEF, %	44.2±10.2	62.0±4.8	0.008

Notes: BMI, body mass index; HbA1c, glycosylated hemoglobin; LDL-C, low-density lipoprotein cholesterol; HDL-C, high-density lipoprotein cholesterol; BNP, brain natriuretic peptide; LVEDD, left ventricular end diastolic diameter; LVEF, left ventricular ejection fraction.

**Table 2:** The 30 highly expressed protein-coding and non protein-coding transcripts in EAT in CAD patients.

Track ID	Gene Name	Transcript Type	Length(bp)	Protein
ENST00000165086.8	NPIPB4	Processed transcript	464	No
ENST00000173785.4	KLF6	Processed transcript	925	No
ENST00000214893.9	ERMP1	Processed transcript	4974	No
ENST00000216463.8	TIMM9	Processed transcript	1075	No
ENST00000216520.6	ERH	Processed transcript	668	No
ENST00000217890.10	ARSD	Processed transcript	2160	No
ENST00000230914.4	MRPS30	Processed transcript	4331	No
ENST00000233699.8	POLE4	Processed transcript	602	No
ENST00000237177.10	CASP8AP2	Processed transcript	6719	No
ENST00000244070.7	PPP4R1L	Processed transcript	1474	No
ENST00000253320.8	TXLNGY	Retained intron	7299	No
ENST00000254109.9	CLUHP3	Processed transcript	1812	No
ENST00000254299.8	GCH1	Processed transcript	2901	No
ENST00000256692.5	PLEKHA8P1	Processed transcript	1839	No
ENST00000263511.8	CROCCP3	Processed transcript	5368	No
ENST00000264785.11	WDR1	Processed transcript	549	No
ENST00000265450.5	TSPAN14	Processed transcript	2588	No
ENST00000265870.7	SLC25A16	Processed transcript	2295	No
ENST00000267869.8	GTF2A2	Processed transcript	518	No
ENST00000267918.9	ANP32A	Processed transcript	1084	No
ENST00000273411.2	RPL39P5	Processed transcript	449	No
ENST00000274820.7	RPL13P5	Processed transcript	349	No
ENST00000276096.10	EBP	Processed transcript	904	No
ENST00000282943.9	ADGRA3	Processed transcript	3534	No
ENST00000286777.6	RWDD2B	Processed transcript	1625	No
ENST00000294661.8	C1orf52	Processed transcript	3391	No
ENST00000295549.8	LINC01116	lincRNA	1407	No
ENST00000295748.7	AZI2	Processed transcript	3127	No
ENST00000296031.4	CXCL2	Processed transcript	577	No
ENST00000296325.9	LRPAP1	Processed transcript	1078	No
ENST00000361624.2	MT-CO1	Protein coding	1542	513aa
ENST00000362079.2	MT-CO3	Protein coding	784	261aa
ENST00000361390.2	MT-ND1	Protein coding	956	318aa
ENST00000361381.2	MT-ND4	Protein coding	1378	459aa
ENST00000361453.3	MT-ND2	Protein coding	1042	347aa
ENST00000361789.2	MT-CYB	Protein coding	1141	380aa
ENST00000361739.1	MT-CO2	Protein coding	684	227aa
ENST00000361851.1	MT-ATP8	Protein coding	207	68aa
ENST00000331825.11	FTL	Protein coding	871	175aa
ENST00000361335.1	MT-ND4L	Protein coding	297	98aa
ENST00000361567.2	MT-ND5	Protein coding	1812	603aa
ENST00000361227.2	MT-ND3	Protein coding	346	115aa
ENST00000361899.2	MT-ATP6	Protein coding	681	226aa
ENST00000320868.9	HBA1	Protein coding	577	142aa
ENST00000331523.6	EEF1A1	Protein coding	1923	462aa
ENST00000327726.10	CFD	Protein coding	1201	253aa
ENST00000302754.5	JUNB	Protein coding	1830	347aa
ENST00000239938.4	EGR1	Protein coding	3137	543aa
ENST00000356524.9	SAA1	Protein coding	518	122aa
ENST00000633942.1	PLIN4	Protein coding	6484	1372aa
ENST00000367029.5	G0S2	Protein coding	876	103aa
ENST00000309311.7	EEF2	Protein coding	3158	858aa
ENST00000251595.11	HBA2	Protein coding	576	142aa
ENST00000335295.4	HBB	Protein coding	628	147aa
ENST00000256104.4	FABP4	Protein coding	941	132aa
ENST00000451311.7	TMSB4X	Protein coding	622	44aa
ENST00000233143.6	TMSB10	Protein coding	461	44aa
ENST00000330871.3	SOCS3	Protein coding	2734	225aa
ENST00000336615.9	PNPLA2	Protein coding	2416	504aa
ENST00000300055.10	PLIN1	Protein coding	2916	522aa

Note: EAT, epicardial adipose tissue; CAD, coronary artery disease; Track ID, The transcript name in Ensembl database; Gene Name, The corresponding gene name of transcript; Transcript Type, the biotype of transcript; Protein, the residue number of protein.

**Table 3:** Differentially expressed lncRNA in EAT in CAD patients with HF compared with CAD patients without HF.

lncRNA	Type	Regulation	Gene Name	Fold Change	P Value
ENST00000610659	exon sense-overlapping	Up	UNC93B1	4.778	0.0118
ENST00000379935	natural antisense	Up	RBL2	3.711	0.0347
ENST00000603195	exon sense-overlapping	Up	ZSWIM8	3.329	0.0039
ENST00000439904	exon sense-overlapping	Up	SLC25A16	2.955	0.0179
ENST00000622120	intergenic	Up	LINC00963	2.952	0.0000
ENST00000514805	exon sense-overlapping	Up	TRIM52	2.901	0.0294
ENST00000492356	exon sense-overlapping	Up	RPS21	2.869	0.0461
ENST00000394225	exon sense-overlapping	Up	NDUFC1	2.840	0.0331
ENST00000548989	exon sense-overlapping	Up	CRIP2	2.247	0.0248
ENST00000465589	exon sense-overlapping	Up	OBSL1	2.229	0.0067
ENST00000398078	exon sense-overlapping	Up	PDXK	2.111	0.0157
ENST00000476113	exon sense-overlapping	Up	TCEA2	2.111	0.0223
ENST00000421064	natural antisense	Up	AP000347.2	2.099	0.0220
ENST00000470322	exon sense-overlapping	Up	ACTR1A	2.083	0.0304
ENST00000587762	intergenic	Up	AC020916.1	2.054	0.0443
ENST00000512955	exon sense-overlapping	Up	AMOTL2	2.052	0.0164
ENST00000508948	exon sense-overlapping	Up	ARRDC3	2.012	0.0289
ENST00000543826	exon sense-overlapping	Down	ADGRD1	0.161	0.0021
ENST00000467318	exon sense-overlapping	Down	DDX56	0.243	0.0126
ENST00000556961	exon sense-overlapping	Down	FBLN5	0.251	0.0033
ENST00000505923	exon sense-overlapping	Down	WDFY3	0.283	0.0221
ENST00000578571	exon sense-overlapping	Down	PTPRM	0.305	0.0360
ENST00000427261	intergenic	Down	RP11-640M9.2	0.331	0.0051
ENST00000319685	exon sense-overlapping	Down	TMTC1	0.370	0.0256
ENST00000493951	exon sense-overlapping	Down	TACC2	0.391	0.0341
ENST00000480603	exon sense-overlapping	Down	PPIA	0.414	0.0048
ENST00000345896	exon sense-overlapping	Down	CERS2	0.453	0.0391
ENST00000498053	exon sense-overlapping	Down	LRRFIP1	0.475	0.0350
ENST00000467178	exon sense-overlapping	Down	CIZ1	0.486	0.0448
ENST00000468975	exon sense-overlapping	Down	ARFGAP1	0.495	0.0128

Note: EAT, epicardial adipose tissue; CAD, coronary artery disease; HF, heart failure; lncRNA, The lncRNA name in Ensembl database; Type, the type of lncRNA; Regulation, the regulation expression of lncRNA; Gene Name, The corresponding gene name of lncRNA.

## Data Availability

The data used to support the findings of this study are available from the corresponding author upon request.
